# Antimicrobial Resistance Patterns in Patients with Vaginal Discharge: A 2019-2022 Analysis at the National Health Laboratory in Eritrea

**DOI:** 10.1155/2024/7193490

**Published:** 2024-03-28

**Authors:** Khalid Hussein, Berhe Tesfai, Okbu Frezgi, Hagos Hayelom, Yosan Gebremeskel, Azania Werede, Hailemichael Gebremariam, Fitsum Kibreab, Mohammed Elfatih Hamida

**Affiliations:** ^1^Department of Obstetrics and Gynecology, Orotta College of Medicine and Health Sciences, Orotta National Referral Maternity Hospital, Ministry of Health, Asmara, Eritrea; ^2^Microbiology Department, National Health Laboratory, Ministry of Health, Asmara, Eritrea; ^3^National Medicines and Food Administration, Ministry of Health, Asmara, Eritrea; ^4^Dekemhare Hospital, Zoba Debub, Ministry of Health, Dekemhare, Eritrea; ^5^Ministry of Health, Division of the Center for Health Research and Resources, Asmara, Eritrea; ^6^Unit of Medical Microbiology, Orotta College of Medicine and Health Sciences, Asmara, Eritrea

## Abstract

**Background:**

Antimicrobial resistance poses a significant global threat to the treatment of bacterial infections, particularly in low- and middle-income regions such as Africa. This study is aimed at analyzing antimicrobial resistance patterns in vaginal swab samples from patients at the National Health Laboratory from 2019 to 2022.

**Methods:**

This retrospective study examined patient records from vaginal swab analyses performed at the National Health Laboratory between January 1, 2019, and December 31, 2022. Ethical approval was obtained from the Ministry of Health Research Ethical Approval and Clearance Committee on 15/02/2023.

**Results:**

Of the 622 samples, 83% underwent microbial isolation and identification. *Citrobacter* spp. exhibited high resistance (>43%) to antibiotics such as cephalexin, ceftazidime, nalidixic acid, ampicillin, gentamicin, and tetracycline. *E. coli* showed resistance rates of more than 50% to ampicillin, trimethoprim-sulfamethoxazole, and tetracycline. *Klebsiella* spp. and Proteus spp. exhibited resistance rates that exceeded 47% to specific antibiotics. Gram-positive bacteria have resistance rates of more than 49% with ampicillin, trimethoprim-sulfamethoxazole, tetracycline, oxacillin, vancomycin, and penicillin G. In particular, *S. aureus* demonstrated no resistance to rifampicin or clindamycin, while *Streptococcus* spp. showed 100% resistance to rifampicin and vancomycin. Several species, including *Proteus* species, *Streptococcus* spp., *S. aureus*, and *Klebsiella* spp. exhibited multidrug resistance.

**Conclusion:**

Most gram-negative bacteria displayed higher resistance of >45% to ampicillin, trimethoprim-sulfamethoxazole, and tetracycline. Among gram-positive bacteria, a higher resistance rate with ampicillin, trimethoprim-sulfamethoxazole, tetracycline, oxacillin, vancomycin, and penicillin G was recorded. *S. aureus* showed no resistance to rifampicin and clindamycin, and *Strep.* spp. indicated 100% resistance to rifampicin and vancomycin. This study highlights critical gaps and areas for further exploration. Expanding the spectrum of antibiotics tested and investigating underlying multidrug resistance mechanisms would provide a more comprehensive understanding of resistance patterns.

## 1. Introduction

The vagina is a complex ecosystem containing a variety of microorganisms [1]. This unique environment undergoes significant changes throughout life, from birth to puberty and menopause [2]. Women are more prone to urinary and vaginal infections due to their anatomical and functional proximity to their anal canal and due to the short urethra [3, 4]. The vaginal area is considered a complex microbial environment that harbors a multitude of microbial species [5]. A common cause of vaginal discharge in women is bacterial vaginosis. Various rod-shaped gram-positive and gram-negative bacteria, including *E. coli*, *Klebsiella* spp., *Enterococcus* spp., *Enterobacter* spp., and *Staphylococcus* spp., contribute significantly to bacterial vaginosis [6, 7].

Vaginal discharge is a common symptom seen by clinicians. It may be physiological or pathological. Normal physiological discharge changes with the menstrual cycle. It is thick and sticky for most of the cycle but becomes clearer, wetter, and stretchy for a short period around the time of ovulation. However, abnormal vaginal discharge is characterized by a change in color, consistency, volume, or odor and may be associated with symptoms such as itch, soreness, dysuria, pelvic pain, or intermenstrual or postcoital bleeding [8].

Pathologic vaginal discharges are caused by a variety of infectious and noninfectious causes. Discharge may be caused by infections of the vagina itself, but infections or inflammation of the cervix also leads to increased vaginal discharge [9]. About 75% of women have the risk of vaginal infections at least once during their lifetime, and the vaginal tract can be infected by common pathogens, including *Enterobacteriaceae* spp., *Enterococcus* sp., and *Streptococcus* sp., *Staphylococcus* sp., *Lactobacillus* sp., and *Candida albicans* [5].

Antimicrobial resistance (AMR) is defined as the inherited or acquired ability of a microorganism to stop the antimicrobial drug from working against it to the extent that it cannot be used any longer [10]. In the last two decades, multi-drug-resistant bacteria have increased and the number of pharmaceutical companies developing new antimicrobial agents has declined [11]. Antibiotic susceptibility testing remains the standard diagnostic method for detecting bacterial resistance and guides clinicians in the appropriate and timely treatment of bacterial infections [12]. The growing multidrug resistance of *gonococci* and the absence of an antibiotic regimen that is shown to be optimal in terms of safety and effectiveness are a challenge for almost every health system [13].

Pathogenic bacteria colonize the birth canal primarily after fecal contamination [14] and are then sometimes transmitted to the baby during labor and delivery [15]. This transmission is probably one of the main sources of neonatal bacterial infection within the first week of life, particularly if there was prolonged/blocked labor or premature rupture of the membranes [16–18]. Vaginal swabs were obtained from 50 individuals with symptoms of vaginal discharge. Bacterial isolates include 20 (43.4%) of *E. coli*, 8 (17.3%) of *Klebsiella* spp., and 8 (17.3%) of *Staphylococcus* spp. The *E. coli*, *Staphylococcus* spp., *Enterobacter* spp., *E. faecalis*, and *R. ornithinolytica* isolates were found resistant to several antibiotics and considered multidrug resistance (MDR) [6]. The most common pathogens that cause vaginal discharge are *Chlamydia trachomatis*, *Neisseria gonorrhoeae*, *Trichomonas vaginalis*, and *Mycoplasma genitalium* [19, 20].

According to a study conducted in Ethiopia, *S. aureus* was a highly prevalent bacteria and resistant to erythromycin (69.8%), trimethoprim/sulfamethoxazole (53.5%), and amoxicillin (39.5%), but susceptible to ciprofloxacin (93%), gentamicin (93%), and clindamycin (81.4%). Of the gram-negative bacteria, *E. coli* was a highly prevalent bacteria and resistant to trimethoprim/sulfamethoxazole (91.3%) and ceftriaxone (63.6%), but susceptible to ciprofloxacin (95.5%), gentamicin (93%), and nitrofurantoin (81.8%) [11].

In a study conducted in Eritrea, the most common isolates of surgical site infection were *Citrobacter* spp., *Klebsiella* spp., *E. coli*, *Proteus* spp., *Pseudomonas aeruginosa*, *Salmonella* spp., *Enterobacter* spp., and *Acinetobacter* spp. The predominant gram-positive bacteria include *S. aureus*, *CONS*, and *Streptococcus viridans* [21]. The *E. coli* isolates exhibited high resistance (>60% resistance) to chloramphenicol, ciprofloxacin, trimethoprim-sulfamethoxazole, gentamicin, nitrofurantoin, tetracycline, cephalexin, ceftazidime, and ceftriaxone. Similarly, *Klebsiella* spp. exhibited high resistance (>60%) to nitrofurantoin, cephalexin, ceftazidime, and ceftriaxone. The isolate had >70% resistance to various agents, including tetracycline, cephalexin, ceftazidime, and ceftriaxone [21].

There is no data repository on AMR-related research on humans, animals, food, plants, and environment isolates in Eritrea, and, as such, it is difficult to estimate the national health and economic impact of AMR [22]. The availability of updated epidemiological data on antimicrobial resistance in frequently encountered bacterial pathogens will be useful not only for deciding on treatment strategies but also for designing an effective antimicrobial stewardship program in hospitals. Therefore, this study was carried out to evaluate the bacterial pathogens involved in vaginal discharge and their antimicrobial susceptibility pattern in patients referred to the National Health Laboratory (NHL) Microbiology Department from 2019 to 2022. The study included the drugs listed on the Eritrean National List of Medicines (ENLM) that are available in the country according to the government policy corresponding to the Clinical Laboratory Standard Institute (CLSI) guidelines, in order to provide baseline information on sensitivity to these drugs. Furthermore, this study is expected to make a great contribution as it could be a baseline for further research and will help access the common cause of vaginal discharge and its pattern of antibiotic resistance, which may help to design a strategy for effective and proper use of antimicrobial drug use.

## 2. Materials and Methods

### 2.1. Study Design

This study used a retrospective study design, conducting a comprehensive review of patient registry records for vaginal swab analyses performed at the National Health Laboratory in Eritrea from January 1, 2019, to December 31, 2022.

### 2.2. Study Population

The study population included all patients who underwent vaginal swab analysis at the National Health Laboratory during the specified study period, from various hospitals in Eritrea.

### 2.3. Sample Size and Sampling Procedure

To ensure a representative dataset, we used a census sampling approach, which included all available laboratory records of patients with complete data within the study period. This methodology was chosen to minimize selection bias and provide a comprehensive overview of antimicrobial resistance patterns in the study population.

### 2.4. Inclusion and Exclusion Criteria

The inclusion criteria comprised all laboratory records of patients who underwent vaginal swab analysis during the study period and who had complete and nonduplicated information. Patients with incomplete or duplicate records were systematically excluded from the study. These criteria were established to maintain the integrity of the data and ensure the validity of the analysis.

### 2.5. Data Collection

A specific data extraction tool was meticulously designed to retrieve essential information from the laboratory record. Skilled laboratory personnel, specifically trained for this study, were responsible for data collection to ensure data accuracy and reliability. Before full-scale data extraction, a pilot study was conducted to validate the data collection tool. This pilot study not only confirmed the effectiveness but also provided information on its refinement. Adjustments were made to the context and objectives of the study findings based on the pilot study.

The data collection tool included critical variables, including the patient's age, the year of analysis, the isolated pathogen, and the resistance pattern exhibited by the isolated organism. Resistance patterns were classified according to the established criteria to ensure consistency in data interpretation.

### 2.6. Ethical Considerations

Ethical approval for this retrospective study was obtained from the Ministry of Health, State of Eritrea, Research Ethics Approval and Clearance Committee (no.: 15/02/2023). Throughout the data collection process, strict measures were implemented to protect patient confidentiality and informed consent procedures were followed as required by ethical guidelines.

### 2.7. Laboratory Procedure

#### 2.7.1. Collecting Clinical Samples and Identification of Pathogens

The genital specimen was inoculated on chocolate agar, MacConkey agar, mannitol salt, and Thayer-Martin and Sabouraud chloramphenicol agar plates. Gram staining was done on the original samples. The chocolate and Thayer-Martin plates were incubated in a jar with a CO_2_ generation kit, and the MacConkey, mannitol salt, and Sabouraud chloramphenicol agar plates were incubated aerobically at 35-37°C for 18-24 hours. Plates were examined after incubation based on their morphology, size, color, consistency, and number of colonies. Gram staining was performed to verify the predominant organisms and subgroup the colonies into gram-negative and gram-positive groups.

The appropriate biochemical tests were then performed to help identify the suspected bacteria, and the appropriate sensitivity tests were performed. Finally, with the help of the growth characteristic of each plate and/or with the result of biochemical tests (catalase tests, deoxyribonuclease (DNase test), triple sugar iron (TSI), Simmons citrate, urease test, tryptophan deamination (TDA), methyl red (MR), indole, amino acid decarboxylation tests, and carbohydrate fermentation tests) and with the help of the numerical identification of the reference book using the API 20E system [23], the possible identification results and the sensitivity result were stated and documented.

#### 2.7.2. Candida albicans and Yeast Detection

Candida albicans and yeast cells were obtained by cultivating suspected samples on Sabouraud chloramphenicol agar (SCA) and subsequently confirmed morphologically using filamentation test methods.

#### 2.7.3. Antibiotic Susceptibility Test (AST)

Antimicrobial susceptibility was achieved using the Kirby-Bauer disc diffusion method (CLSI modified disc diffusion technique) on the Mueller-Hinton agar. Briefly, the turbidity of the bacteria was measured by comparing the pure colonies emulsified with normal saline and 0.5 McFarland solution. The bacteria were susceptible to ampicillin (Amp) (10 *μ*g), gentamicin (GEN) (10 *μ*g), co-trimoxazole (COT) (25 *μ*g), erythromycin (ERY) (15 *μ*g), amikacin (AMK) (30 *μ*g), ceftazidime (CAZ) (30 *μ*g), penicillin (PEN) (10 IU), tetracycline (TET) (30 *μ*g), nalidixic acid (NAL) (30 *μ*g), ciprofloxacin (CIP) (5 *μ*g), chloramphenicol (CHL) (30 *μ*g), cephalexin (CL) (30 *μ*g), ceftriaxone (CRO) (30 *μ*g), nitrofurantoin (F) (300 *μ*g), clindamycin (CD) (2 *μ*g), oxacillin (ox) (1 *μ*g), rifampicin (Rif) (5 *μ*), and vancomycin (Van) (30 *μ*g). The isolated bacteria were seeded on a dry Mueller-Hinton agar plate with appropriate antimicrobial impregnated disks and cultured overnight at 35°C-37°C. Antibiotic inhibition zones were measured from the center to the different edges of the antibiotic inhibition zones using a ruler. The AST discs were obtained from Thermo Scientific™ Oxoid™.

### 2.8. Quality Control

The strains E. coli ATCC 25922, S. aureus ATCC 25923, and E. faecalis ATCC 29212 were used as control organisms to check the performance of the media and discs. They were tested for quality control after each microbiological procedure, such as staining, antimicrobial susceptibility tests, and biochemical identification procedures once a month and before the use of a new batch of reagents and antimicrobials.

### 2.9. Data Analysis

The completeness of the collected data was further checked for completeness prior to data entry. Finally, the data were entered in MS Excel and further exported to SPSS version 25 for analysis. The frequency and percentage were determined. After collecting the results, some of the bacteria were grouped into their species for analysis as follows: *Citrobacter* spp. (*Citrobacter diversus*, *Citrobacter freundii*), coagulase-negative *Staphylococcus* (*CONS*) (*S. epidermidis*, *S. saprophyticus*), other gram negative (*Aeromonas* spp., *Ent. aerogenes*, *Enterobacter cloacae*, *Enterobacter agglomerans*, *Kluyvera* spp., *Morganella morganii*, *Pseudomonas* spp., and *Ser. liquefaciens*), *Klebsiella* spp. (*K. oxytoca*, *K. pneum. ozaenae*, and *K. pneum. pneumoniae*), nongroupable *Streptococcus* (*S. viridans*), *Proteus* spp. (*Proteus mirabilis*, *Proteus vulgaris*), and *Streptococcus* spp. (*Strep. group B*, *streptococcal group D*, and *S. pneumoniae*).

### 2.10. Ethical Considerations

Ethical approval was obtained from the Ministry of Health Research Ethical Approval and Clearance Committee on 15/22/203. The head of the National Health Laboratory was informed, and permission was sought from the authorities. Since these were secondary data, informed consent was not sought, but confidentiality of the patient's laboratory records was maintained secure and the patient's personal identification was coded and analyzed as aggregates.

## 3. Results

### 3.1. Characteristics of Microbial Isolates from Vaginal Discharge in National Health Laboratory

During the study period, 622 vaginal samples were cultured in the NHL, and 515/622 were infected with microorganisms, a positivity rate of 83%. Most of the patients were between 18 and 35 years old, 431 (61.9%) and 11 of the patients were under 6 years old, and 36% of the cases were investigated in 2022 (Table 1).

In addition, the 170 (33%) isolates from the positive results were pathogenic bacteria and antimicrobial sensitivity was done for them. The most predominant of these isolated bacteria were *E. coli* 87 (51.2%) followed by *S. aureus* 27 (15.9%), *Klebsiella* spp. 19 (11.2%), other gram negative 14 (8.2%), *Streptococcus* spp. 10 (5.9%), *Citrobacter* spp. 7 (4.1%), and *Proteus* spp. 6 (3.5%). The remaining 345 (67%) isolates were not clinically significant microorganisms (Table 1).

### 3.2. Antimicrobial Sensitivity to Different Antibiotics from Vaginal Swab Isolates in the National Health Laboratory

Of the isolated pathogenic microorganisms, they were sensitive to rifampicin 27 (84.4%), chloramphenicol 141 (82.9%), ceftriaxone 105 (78.9%), ceftazidime 104 (73.2%), ciprofloxacin 123 (74.4%), and nitrofurantoin 120 (74.4%). And some isolates were resistant to penicillin G 24 (80.8%), ampicillin 98 (71.0%), oxacillin 18 (56.3%), tetracycline 88 (51.2%), trimethoprim-sulfamethoxazole (co-trimoxazole) 78 (48.5%), and vancomycin 20 (54.1%) (Table 2).

### 3.3. Antimicrobial Sensitivity Based on Specific Bacterial Isolates from Vaginal Swab in National Health Laboratory (Gram-Negative Species)

**Figure 1:**
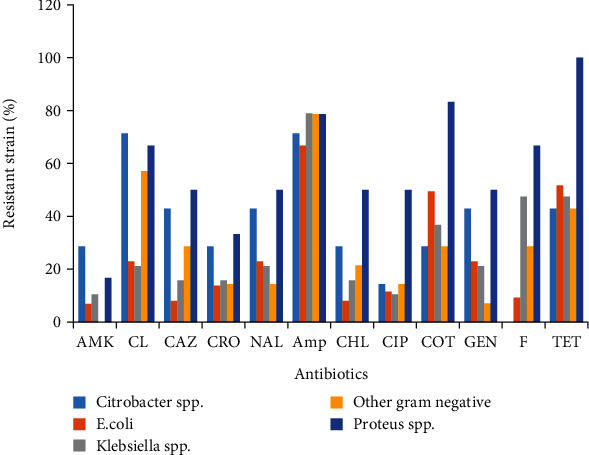
Antibiotic resistance patterns of gram-negative bacteria. Amp: ampicillin; GEN: gentamicin; COT: co-trimoxazole; ERY: erythromycin; AMK: amikacin; CAZ: ceftazidime; PEN: penicillin; TET: tetracycline; NAL: nalidixic acid; CIP: ciprofloxacin; CHL: chloramphenicol; CL: cephalexin; CRO: ceftriaxone; F: nitrofurantoin; CD: clindamycin; ox: oxacillin; Rif: rifampicin; Van: vancomycin.

A total of 12 antibiotics including amikacin, cephalexin, ceftazidime, ceftriaxone, nalidixic acid, ampicillin, chloramphenicol, ciprofloxacin, trimethoprim-sulfamethoxazole, gentamicin, nitrofurantoin, and tetracycline were tested in gram-negative bacteria. The result showed that no *Citrobacter* spp. was resistant to nitrofurantoin and also no other gram-negative bacteria were resistant to amikacin. Citrobacter spp. recorded the highest resistance rate (>43%) with cephalexin, ceftazidime, nalidixic acid, ampicillin, gentamicin, and tetracycline. For *E. coli*, the highest resistance rates were recorded (>50%) with ampicillin, trimethoprim-sulfamethoxazole, and tetracycline. Furthermore, the lowest resistance rates (<9%) were recorded with amikacin, ceftazidime, chloramphenicol, and nitrofurantoin. In *Klebsiella* spp., the highest resistance rates (>47%) were recorded with ampicillin, nitrofurantoin, and tetracycline and the lowest resistance rates (<11%) for this strain were recorded with amikacin and ciprofloxacin. For *Proteus* spp., the resistance rates were more than 50% for almost all antibiotics tested. In the other gram-negative bacteria, high resistance rates (>57%) were recorded with cephalexin and ampicillin and the lowest resistance rates (<14%) were recorded with ceftriaxone, nalidixic acid, ciprofloxacin, and gentamicin. Of all isolated gram-negative bacteria, the highest resistance rates (>46%) were recorded with ampicillin and tetracycline. However, the lowest resistance rates (15%) with these bacteria were recorded with amikacin, ceftazidime, and ceftriaxone (Table 3 and Figure 1).

### 3.4. Antimicrobial Sensitivities of Vaginal Swab in National Health Laboratory (Gram-Positive Species)

**Figure 2:**
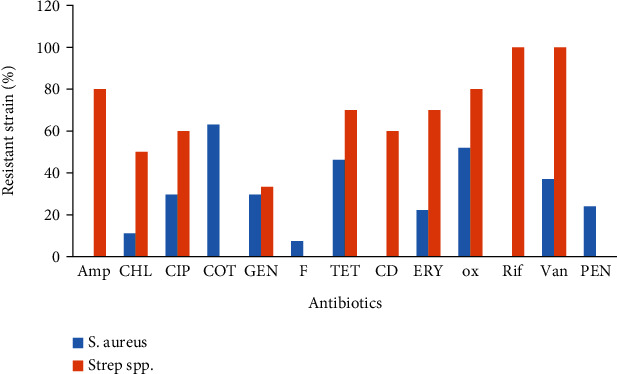
Antibiotic resistance patterns of gram-positive bacteria. Amp: ampicillin; CHL: chloramphenicol; CIP: ciprofloxacin; COT: co-trimoxazole; GEN: gentamicin; F: nitrofurantoin; TET: tetracycline; CD: clindamycin; ERY: erythromycin; ox: oxacillin; Rif: rifampicin; Van: vancomycin; PEN: penicillin.

A total of 13 antibiotics including ampicillin, chloramphenicol, ciprofloxacin, trimethoprim-sulfamethoxazole, gentamicin, nitrofurantoin, tetracycline, clindamycin, erythromycin, oxacillin, rifampicin, vancomycin, and penicillin G were tested for gram-positive bacteria. Of all isolated gram-positive bacteria, the highest resistance rates (>49%) were recorded with ampicillin, trimethoprim-sulfamethoxazole, tetracycline, oxacillin, vancomycin, and penicillin G. and the lowest resistance rates (<17%) were recorded with chloramphenicol, ciprofloxacin, nitrofurantoin, clindamycin, and rifampicin. No *S. aureus* resistant to rifampicin and clindamycin was found. With those strains of bacteria, the highest resistance rates (>46%) were recorded with trimethoprim-sulfamethoxazole, tetracycline, oxacillin, and penicillin G. The lowest resistance rates (<11%) were recorded with chloramphenicol and nitrofurantoin. In the case of *Strep.* spp., antimicrobial resistance testing included ampicillin, chloramphenicol, ciprofloxacin, gentamicin, tetracycline, clindamycin, erythromycin, oxacillin, rifampicin, and vancomycin. The result revealed that it was 100% resistant to rifampicin and vancomycin. And the resistance rate was more than 50% for ampicillin, chloramphenicol, ciprofloxacin, tetracycline, clindamycin, erythromycin, and oxacillin. The lowest resistance rates (33%) were recorded with gentamicin (Table 4 and Figure 2).

### 3.5. Multidrug Resistance of Gram-Negative and Gram-Positive Isolates from Vaginal Discharge

Most species showed multidrug resistance as *Proteus* species, *Streptococcus* spp., *S. aureus*, and *Klebsiella* spp. as R1 (single drug resistance), R2 (double drug resistance), and R3-R9 (multidrug resistance) (Table 5).

## 4. Discussion

Combating antimicrobial resistance is crucial, especially in developing countries like Eritrea. This can add a burden to different preexisting challenges such as misuse of medications, availability, and higher level of substandard medications. Another study revealed that in developing countries, the high proportion of life-threatening bacterial infections, exacerbated by inadequate awareness, laboratory facilities, and human resources for health, is expected to worsen the impact of AMR. Similarly, different studies reported that self-medication, empirical therapy, misuse, and overuse of antimicrobials increase antimicrobial resistance and lead to prolonged illness, disability, increased health care costs, and death [24]. Enhancing community awareness and health professionals can stop the growth of antimicrobial resistance.

The microbial growth rate was 83%, and the bacteria that dominated the most were *E. coli* (51.2%) and *S. aureus* (15.9%). This was consistent with the previous study in which *E. coli* (43.4%), *Klebsiella* spp. (17.3%), and *Staphylococcus* spp. (17.3%) were the most common isolates [6]. Another study showed that the most prevalent pathogen was *E. coli*, *Klebsiella pneumoniae*, *Staphylococcus aureus*, and *Citrobacter* spp. [25]. Furthermore, a study conducted in Eritrea showed that the most common isolates included *Citrobacter* spp., *Klebsiella* spp., *E. coli*, *Proteus* spp., *S. aureus*, *CONS*, and *Streptococcus viridans*, although the sample and frequency order were different [21]. However, it was inconsistent with a study that 22.7% of isolates were *G. vaginalis*, and the predominant aerobic bacteria were *S. aureus* (25.4%), *S. agalactiae* (14.1%), and *E. coli* (13.5%) [11].

This study revealed that 11 of the patients were under six years old and six of these samples showed bacterial growth and antimicrobial susceptibility was done. Another study reported that a total of 99 samples were collected from children, of which 78 (30.4%) were culture positive [26]. Clinically, different articles in the literature reported that vaginal discharge is not a common presentation in this age group and bacterial growth is not commonly expected [26]. These children could have any predisposing factors such as immune suppressive diseases and malnutrition and need further investigation to determine the primary cause.

The antimicrobial susceptibility assay revealed a variable resistance pattern to penicillin G, ampicillin, trimethoprim-sulfamethoxazole, tetracycline, and oxacillin. And most were sensitive to ceftriaxone, ceftazidime, chloramphenicol, ciprofloxacin, nitrofurantoin, and rifampicin. This was inconsistent with a study in which clindamycin (40%), ampicillin (27%), vancomycin (26%), ciprofloxacin (18%), and nitrofurantoin (12%) showed the highest resistance to surgical site infections in Eritrea [21]. This could be mainly due to the type of sample, and commonly used medications can differ for the indication of treating vaginal discharge, which can result in various patterns of drug resistance.

Gram-negative bacteria such as *Citrobacter* spp., *E. coli*, *Klebsiella* spp., and *Proteus* spp. were resistant to ampicillin. Similarly, another study showed that *E. coli* was resistant to trimethoprim/sulfamethoxazole and ceftriaxone but susceptible to ciprofloxacin, gentamicin, and nitrofurantoin [11]. Ampicillin is one of the antibiotics commonly prescribed for gram-positive and gram-negative bacteria that can develop resistance, but it is not among the medications commonly prescribed for patients with vaginal discharge.

*Citrobacter* spp. were resistant to ampicillin and cephalexin, which coincides with other studies showing that *Citrobacter* spp. isolates had >70% resistance to various agents, including tetracycline, cephalexin, ceftazidime, and ceftriaxone [21]. This group of gram-negative bacteria is not among the common causes of vaginal discharge but was isolated in a previous study from surgical site infections in Eritrea [21]. *Klebsiella* spp. were resistant to ampicillin, tetracycline, nitrofurantoin, and trimethoprim-sulfamethoxazole. Similarly, studies reported that Klebsiella spp. exhibited high resistance to ampicillin and trimethoprim-sulfamethoxazole [6] and isolates exhibited high resistance (>60%) to nitrofurantoin, cephalexin, ceftazidime, and ceftriaxone [21]. This could be mainly due to the fact that these antibiotics are among the antibiotics commonly prescribed for these gram-negative bacteria for different diseases.

The *Proteus* species were resistant to tetracycline, ampicillin, trimethoprim-sulfamethoxazole, cephalexin, ceftazidime, nalidixic acid, nitrofurantoin, gentamicin, chloramphenicol, and ciprofloxacin. Proteus vulgaris showed a sensitivity to ciprofloxacin (87.0%). *Diphtheroids* showed sensitivity to ampicillin (95.7%) and ceftriaxone (91.3%) [27]. These bacteria showed a high degree of antimicrobial resistance to most medications that could be a threat to the community and health professionals.

*Escherichia coli* was resistant to ampicillin, tetracycline, and trimethoprim-sulfamethoxazole but was highly sensitive to chloramphenicol, ceftazidime, nitrofurantoin, ceftriaxone, and ciprofloxacin. Similarly, *E. coli* was highly resistant to ampicillin, cefazolin, and trimethoprim-sulfamethoxazole [6], and *E. coli* were resistant to trimethoprim/sulfamethoxazole and ceftriaxone, but susceptible to ciprofloxacin, gentamicin, and nitrofurantoin [11]. Also, *E. coli* isolates exhibited high resistance (>60% resistance) to chloramphenicol, ciprofloxacin, trimethoprim-sulfamethoxazole, gentamicin, nitrofurantoin, tetracycline, cephalexin, ceftazidime, and ceftriaxone [21]. Based on a study conducted in Eritrea, the *E. coli* isolates were sensitive to chloramphenicol, gentamicin, and ceftriaxone, but resistant to ampicillin and cephalexin. Additionally, *E. coli* showed resistance to ampicillin, tetracycline, and trimethoprim-sulfamethoxazole [24].

This study reported that most gram-positive bacteria were resistant to trimethoprim-sulfamethoxazole, tetracycline, ampicillin, oxacillin, vancomycin, and penicillin G. Similarly, another study reported that most gram-positive bacteria were resistant to erythromycin, trimethoprim/sulfamethoxazole, and amoxicillin [11]. This high level of antimicrobial resistance of gram-positive bacteria will have an effect on the use of these antibiotics for the use of other infections.

*Staphylococcus aureus* was 100% sensitive to rifampicin and clindamycin and was highly resistant to penicillin G, trimethoprim-sulfamethoxazole, oxacillin, and tetracycline. This was consistent with other studies that *S. aureus* was highly resistant to tetracycline and trimethoprim-sulfamethoxazole [24] and *S. aureus* was resistant to erythromycin, trimethoprim/sulfamethoxazole, and amoxicillin. Furthermore, all S. aureus isolates were resistant to penicillin in surgical site infection samples [21], and Staphylococcus has a high level of resistance to oxacillin, benzylpenicillin, levofloxacin, nitrofurantoin, trimethoprim-sulfamethoxazole, clindamycin, erythromycin, and tetracycline [6]. *Streptococcus* spp. showed 100% resistance to rifampicin and vancomycin. Furthermore, there is greater resistance to ampicillin, ciprofloxacin, chloramphenicol, tetracycline, clindamycin, erythromycin, and oxacillin. Although these gram-positive bacteria were not among the common causes of vaginal discharge, they had a high level of resistance that could reduce their use for other indications.

Multidrug resistance (MDR) means concomitant resistance to 3 different antimicrobial classes or nonsusceptible to at least one agent in 3 antimicrobial drug classes [21, 28, 29]. In our study, most species (Proteus species, *Streptococcus* spp., *S.* aureus, and Klebsiella spp.) show multidrug resistance. This result coincides with another study conducted on vaginal swabs in which *E. coli*, *Staphylococcus* spp., and *Enterobacter* spp. isolates were found to be resistant to several antibiotics and considered multidrug resistant [6]. Similarly, another study conducted in Eritrea on surgical site infections revealed that *E. coli*, *Klebsiella* spp., and *Citrobacter* spp. isolates had multiple antimicrobial resistances [21]. This higher rate of commonly used broad-spectrum antibiotics will be a threat to the community, physicians, and patients.

The study was not without limitations. This study showed that there were no isolates of common causes of vaginal discharge such as *C. trachomatis* and *N. gonorrhoeae*. This could be mainly due to the fact that these pathogens are fastidious and need special media, a specific collection, transport, and storage system; thus, they may not show growth in the culture medium. Furthermore, since it was a retrospective study, the background characteristics of the patients were not determined, which may show a specific association with antimicrobial resistance. Furthermore, culture and sensitivity for some bacteria (CONS) were not performed, and poor sample collection may also impact the growth of some pathogens. As this was a retrospective study, we did not follow the outcome of the patients. Furthermore, this result does not generalize antimicrobial resistance for the general population, and self-medication and empirical therapy could contribute to antimicrobial resistance.

## 5. Conclusion

Most isolates were sensitive to ceftriaxone, ceftazidime, chloramphenicol, ciprofloxacin, nitrofurantoin, and rifampicin and resistant to ampicillin and penicillin. Most gram-negative bacteria such as *Citrobacter* spp., *E. coli*, *Klebsiella* spp., and *Proteus* spp. were resistant to ampicillin. Additionally, most Proteus spp. were resistant to cephalexin, ampicillin, trimethoprim-sulfamethoxazole, tetracycline, and nitrofurantoin. Furthermore, most of *S. aureus* were sensitive to clindamycin and rifampicin and resistant to penicillin G, while all species of Streptococcus were resistant to rifampicin and vancomycin. *Streptococcus* spp., *Proteus* species, *Klebsiella* spp., and *Staphylococcus* aureus showed multiple antibiotic resistances.

## 6. Recommendations

Enhancing community awareness of the misuse of medications, substandard drugs, and the burden of antimicrobial resistance is crucial to solve this growing concern. Furthermore, the wise use of medications and the prescription of antibiotic-based culture and sensitivity are cornerstones for the life use of antibiotics. Further prospective studies based on vaginal, urine, and blood cultures are highly recommended in conjunction with the Eritrean national action plan to combat antimicrobial resistance through the “One Health” approach, 2021-2025.

## Figures and Tables

**Table 1:** Characteristics of microbial isolates from vaginal discharge in the National Health Laboratory.

Isolates	Frequency	Percent (%)
*Citrobacter* spp.	7	1.12
*E. coli*	87	14
*Klebsiella* spp.	19	3.1
Other gram-negative	14	2.3
*Proteus* spp.	6	1
*S. aureus*	27	4.3
*Streptococcus*	10	1.6
No growth	107	17.2
Nongroupable *Streptococcus*	18	2.9
*CONS*	199	32
*Gram-positive bacilli*	18	2.9
*Candida albicans*	13	2.1
Yeast	97	15.5
Patients age in years		
<18	26	4.2
18-25	205	32.9
26-35	224	36.2
>35	167	26.8
Year of data collection		
2019	102	16.4
2020	115	18.8
2021	140	22.4
2022	225	36.1
Total	622	100

**Table 2:** Antimicrobial sensitivities to different antibiotics from vaginal swab isolates in the National Health Laboratory.

Antibiotics tested	Antimicrobial sensitivity, frequency (percent), *N* (170)			Total *N* (%)
	Intermediate	Resistant	Sensitive	
Amikacin	26 (19.5)	11 (8.3)	96 (72.2)	133 (100.0)
Ampicillin	14 (10.1)	98 (71.0)	26 (18.8)	138 (100.0)
Cephalexin	51 (38.3)	41 (30.8)	41 (30.8)	133 (100.0)
Ceftazidime	9 (6.8)	20 (15.0)	104 (78.2)	133 (100.0)
Ceftriaxone	7 (5.3)	21 (15.8)	105 (78.9)	133 (100.0)
Nalidixic acid	13 (9.8)	32 (24.1)	88 (66.2)	133 (100.0)
Chloramphenicol	3 (1.8)	26 (15.3)	141 (82.9)	170 (100.0)
Ciprofloxacin	13 (7.9)	29 (17.6)	123 (74.5)	165 (100.0)
Co-trimoxazole	7 (4.4)	78 (48.8)	75 (46.9)	160 (100.0)
Gentamicin	23 (13.9)	41 (24.7)	101 (60.8)	166 (100.0)
Nitrofurantoin	13 (8.1)	27 (16.8)	120 (74.5)	161 (100.0)
Tetracycline	5 (3.0)	88 (52.1)	76 (45.0)	169 (100.0)
Clindamycin	8 (25.0)	3 (9.4)	21 (65.6)	32 (100.0)
Erythromycin	9 (24.3)	13 (35.1)	15 (40.5)	37 (100.0)
Oxacillin	3 (9.4)	18 (56.3)	11 (34.4)	32 (100.0)
Penicillin G	0 (0)	24 (88.9)	3 (11.1)	27 (100.0)
Rifampicin	0 (0)	5 (15.6)	27 (84.4)	32 (100.0)
Vancomycin	0 (0)	20 (54.1%)	17 (45.9)	37 (100.0)

**Table 3:** Antimicrobial sensitivity of vaginal swab in National Health Laboratory (gram-negative species).

		Isolate, *n* (%)					Total (*n* = 133)	*P* value
		*Citrobacter* spp. (*n* = 7)	*E. coli* (*n* = 87)	*Klebsiella* spp. (*n* = 19)	Other gram -ve (*n* = 14)	*Proteus* spp. (*n* = 6)		
Amikacin	I	1 (14.3)	15 (17.2)	7 (36.8)	1 (7.1)	2 (33.3)	26 (19.5)	0.11
	R	2 (28.6)	6 (6.9)	2 (10.5)	0 (0.0)	1 (16.7)	11 (8.3)	
	S	4 (57.1)	66 (75.9)	10 (52.6)	13 (92.9)	3 (50.0)	96 (72.2)	

Cephalexin	I	2 (28.6)	39 (44.8)	7 (36.8)	2 (14.3)	1 (16.7)	51 (38.3)	0.014
	R	5 (71.4)	20 (23.0)	4 (21.1)	8 (57.1)	4 (66.7)	41 (30.8)	
	S	0 (0.0)	28 (32.2)	8 (42.1)	4 (28.6)	1 (16.7)	41 (30.8)	

Ceftazidime	I	0 (0.0)	6 (6.9)	2 (10.5)	1 (7.1)	0 (0.0)	9 (6.8)	0.039
	R	3 (42.9)	7 (8.0)	3 (15.8)	4 (28.6)	3 (50.0)	20 (15.0)	
	S	4 (57.1)	74 (85.1)	14 (73.7)	9 (64.3)	3 (50.0)	104 (78.2)	

Ceftriaxone	I	2 (28.6)	3 (3.4)	0 (0.0)	2 (14.3)	0 (0.0)	7 (5.3)	0.060
	R	2 (28.6)	12 (13.8)	3 (15.8)	2 (14.3)	2 (33.3)	21 (15.8)	
	S	3 (42.9)	72 (82.8)	16 (84.2)	10 (71.4)	4 (66.7)	105 (78.9)	

Nalidixic acid	I	1 (14.3)	5 (5.7)	4 (21.1)	2 (14.3)	1 (16.7)	13 (9.8)	0.253
	R	3 (42.9)	20 (23.0)	4 (21.1)	2 (14.3)	3 (50.0)	32 (24.1)	
	S	3 (42.9)	62 (71.3)	11 (57.9)	10 (71.4)	2 (33.3)	88 (66.2)	

Ampicillin	I	1 (14.3)	9 (10.3)	3 (15.8)	1 (7.1)	0 (0.0)	14 (10.1)	0.85
	R	5 (71.4)	58 (66.7)	15 (78.9)	11 (78.6)	11 (78.6)	100 (71.9)	
	S	1 (14.3)	20 (23.0)	1 (5.3)	2 (14.3)	1 (16.7)	25 (18)	

Chloramphenicol	I	0 (0.0)	1 (1.1)	0 (0.0)	0 (0.0)	0 (0.0)	1 (0.8)	0.01
	R	2 (28.6)	7 (8.0)	3 (15.8)	3 (21.4)	3 (50.0)	18 (14)	
	S	5 (71.4)	79 (90.8)	16 (84.2)	11 (78.6)	3 (50.0)	114 (85.7)	

Ciprofloxacin	I	2 (28.6)	5 (5.7)	1 (5.3)	0 (0.0)	0 (0.0)	8 (6)	0.001
	R	1 (14.3)	10 (11.5)	2 (10.5)	2 (14.3)	3 (50.0)	18 (14)	
	S	4 (57.1)	72 (82.8)	16 (84.2)	12 (85.7)	3 (50.0)	107 (80.5)	

Trimethoprim-sulfamethoxazole	I	1 (14.3)	2 (2.3)	2 (10.5)	1 (7.1)	0 (0.0)	6 (4.5)	0.225
	R	2 (28.6)	43 (49.4)	7 (36.8)	4 (28.6)	5 (83.3)	61 (46)	
	S	4 (57.1)	42 (48.3)	10 (52.6)	9 (64.3)	1 (16.7)	66 (49.6)	

Gentamicin	I	1 (14.3)	13 (14.9)	4 (21.1)	1 (7.1)	2 (33.3)	21 (15.8)	0.001
	R	3 (42.9)	20 (23.0)	4 (21.1)	1 (7.1)	3 (50.0)	31 (23)	
	S	3 (42.9)	54 (62.1)	11 (57.9)	12 (85.7)	1 (16.7)	81 (60.9)	

Nitrofurantoin	I	1 (14.3)	6 (6.9)	1 (5.3)	4 (28.6)	1 (16.7)	13 (9.8)	0.001
	R	0 (0.0)	8 (9.2)	9 (47.4)	4 (28.6)	4 (66.7)	25 (19)	
	S	6 (85.7)	73 (83.9)	9 (47.4)	6 (42.9)	1 (16.7)	95 (71.4)	

Tetracycline	I	1 (14.3)	3 (3.4)	0 (0.0)	1 (7.1)	0 (0.0)	5 (3.8)	0.328
	R	3 (42.9)	45 (51.7)	9 (47.4)	6 (42.9)	6 (100.0)	69 (51.9)	
	S	3 (42.9)	39 (44.8)	10 (52.6)	7 (50.0)	0 (0.0)	59 (44.4)	

I: intermediate; R: resistant: S: sensitive.

**Table 4:** Antimicrobial sensitivities of vaginal swab in National Health Laboratory (gram-positive species).

		*S. aureus* (*n* = 27)	*Strep.* spp. (*n* = 10)	Total, *n* (%) (*n* = 37)	*P* value
Ampicillin	I	0 (0.0)	0 (0.0)	14 (10.1)	0.85
	R	0 (0.0)	4 (80.0)	98 (71.0)	
	S	0 (0.0)	1 (20.0)	26 (18.8)	

Chloramphenicol	I	2 (7.4)	0 (0.0)	3 (1.8)	0.01
	R	3 (11.1)	5 (50.0)	26 (15.3)	
	S	22 (81.5)	5 (50.0)	141 (82.9)	

Ciprofloxacin	I	3 (11.1)	2 (40.0)	13 (7.9)	0.001
	R	8 (29.6)	3 (60.0)	29 (17.6)	
	S	16 (59.3)	0 (0.0)	123 (74.5)	

Trimethoprim-sulfamethoxazole	I	1 (3.7)	0 (0.0)	7 (4.4)	0.22
	R	17 (63.0)	0 (0.0)	78 (48.8)	
	S	9 (33.3)	0 (0.0)	75 (46.9)	

Gentamicin	I	1 (3.7)	1 (16.7)	23 (13.9)	0.001
	R	8 (29.6)	2 (33.3)	41 (24.7)	
	S	18 (66.7)	2 (33.3)	101 (60.8)	

Nitrofurantoin	I	0 (0.0)	0 (0.0)	13 (8.1)	0.001
	R	2 (7.4)	0 (0.0)	27 (16.8)	
	S	25 (92.6)	0 (0.0)	120 (74.5)	

Tetracycline	I	0 (0.0)	0 (0.0)	5 (3.0)	0.33
	R	12 (46.2)	7 (70.0)	88 (52.1)	
	S	14 (53.8)	3 (30.0)	76 (45.0)	

Clindamycin	I	8 (29.6)	0 (0.0)	8 (25.0)	0.001
	R	0 (0.0)	3 (60.0)	3 (9.4)	
	S	19 (70.4)	2 (40.0)	21 (65.6)	

Erythromycin	I	7 (25.9)	2 (20.0)	9 (24.3)	0.018
	R	6 (22.2)	7 (70.0)	13 (35.1)	
	S	14 (51.9)	1 (10.0)	15 (40.5)	

Oxacillin	I	3 (11.1)	0 (0.0)	3 (9.4)	0.471
	R	14 (51.9)	4 (80.0)	18 (56.3)	
	S	10 (37.0)	1 (20.0)	11 (34.4)	

Rifampicin	R	0 (0.0)	5 (100.0)	5 (15.6)	0.001
	S	27 (100.0)	0 (0.0)	27 (84.4)	

Vancomycin	R	10 (37.0)	9 (100.0)	20 (54.1)	0.003
	S	17 (63.0)	0 (0.0)	17 (45.9)	

Penicillin G	R	24 (88.9)	—	24 (88.9)	____
	S	3 (11.1)	—	3 (11.1)	

**Table 5:** Multidrug resistance of gram-negative and gram-positive isolates from vaginal discharge.

Multidrugs		*N* (%)	*Citrobacter* spp.	*E. coli*	*Klebsiella* spp.	Other gram -ve	*Proteus* spp.	*S. aureus*	*Strep.* spp.	*P* value
Amp	R_1_	98 (57.0)	5 (71.4)	58 (66.7)	15 (78.9)	11 (78.6)	5 (83.3)	—	4 (40.0)	<0.001
Amp, CL	R_2_	36 (20.9)	5 (71.4)	19 (21.8)	2 (10.5)	6 (42.9)	4 (66.7)	—	—	<0.001
Amp, F	R_2_	22 (12.8)	—	7 (8.0)	8 (42.1)	4 (28.6)	3 (50.0)	—	—	<0.001
Amp, COT, TET	R_3_	49 (28.5)	2 (28.6)	32 (36.8)	6 (31.6)	4 (28.6)	5 (83.3)	—	—	<0.001
COT, PEN, ox	R_3_	11 (6.4)	—	—	—	—	—	11 (40.7)	—	<0.001
Amp, CL, TET, GEN	R_4_	16 (9.3)	2 (28.6)	9 (10.3)	2 (10.5)	1 (7.1)	2 (33.3)	—	—	0.122
Amp, CL, TET, GEN, CHL, CIP, COT, F	R_8_	4 (2.3)	—	—	1 (5.3)	1 (7.1)	2 (33.3)	—	—	<0.001

R_1_: single drug resistance; R_2_: double drug resistance; R_3_-R_9_: multidrug resistance; Amp: ampicillin; GEN: gentamicin; COT: co-trimoxazole; ERY: erythromycin; AMK: amikacin; CAZ: ceftazidime; PEN: penicillin; TET: tetracycline; SXT: trimethoprim-sulfamethoxazole; NAL: nalidixic acid; CIP: ciprofloxacin; CHL: chloramphenicol; CL: cephalexin; CRO: ceftriaxone; F: nitrofurantoin; CD: clindamycin; ox: oxacillin; Rif: rifampicin; Van: vancomycin.

## Data Availability

All datasets used for this study are available from the corresponding author on reasonable request.
